# Therapeutic Rationale for Endotoxin Removal with Polymyxin B Immobilized Fiber Column (PMX) for Septic Shock

**DOI:** 10.3390/ijms22042228

**Published:** 2021-02-23

**Authors:** Hisataka Shoji, Steven M. Opal

**Affiliations:** 1 Division of Emergency and Critical Care Medicine, Toray Medical Co., Ltd, Tokyo 103-0023, Japan; 2 Division of Infectious Diseases, Rhode Island Hospital and Alpert Medical School of Brown University, Providence, RI 02905, USA; steven_opal@brown.edu

**Keywords:** septic shock, endotoxin adsorption, polymyxin B, hemoperfusion, immunomodulation, idiopathic pulmonary fibrosis, COVID-19

## Abstract

Endotoxin removal therapy with polymyxin B immobilized fiber column (PMX) has been clinically applied for sepsis and septic shock patients since 1994. The effectiveness and usefulness of this therapy have been demonstrated for more than a quarter of a century. However, a documented survival benefit has not yet been demonstrable in a large, multicenter, randomized and controlled trial. Following the findings derived from a large sepsis clinical trial with PMX in North America, a new trial is ongoing to determine if PMX has a long-term survival benefit when administered to septic patients. Another approach to support a survival benefit from intervention with PMX is to utilize a detailed analysis available from a large clinical data base. The endotoxin adsorption capacity of PMX columns in vitro and the effectiveness of PMX columns can be further demonstrable in animal models. The capability of PMX and details of its mechanism of action to intervene in the sepsis cascade and impede organ dysfunction in septic patients is not fully understood. The surface antigen expression in monocytes and neutrophils are improved after PMX therapy. Immunomodulatory effects as a result of endotoxin removal and/or other mechanisms of action have been suggested. These effects and other potential immune effects may explain some of the improved effects upon organ dysfunction of sepsis and septic shock patients. Endotoxemia may be involved in the pathophysiology of other diseases than sepsis. A rapid diagnostic method to detect and target endotoxemia could allow us to practice precision medicine and expand the clinical indications of endotoxin removal therapy.

## 1. Introduction

Endotoxin, also known as lipopolysaccharide (LPS), is a central component of the cell wall component of Gram-negative bacteria. Sepsis is a major threat to human health in the elderly, the immune compromised, trauma patients and post-surgical patients. A recent international consensus conference has generated a new way to classify patients to fit into more strictly defined clinical groups.

This classification scheme recognizes the importance of sepsis as an end product of the pathogen-associated molecular patterns (PAMPs) to trigger the host response to infection. The host response is characterized by both proinflammatory and anti-inflammatory responses. A dysregulated host response causes organ dysfunction and leads to poor outcome [1]. Sepsis is now considered as: (1) an acute systemic inflammatory state, driven by a microbial pathogen (2) in which the dysfunctional host response is actually contributing to systemic inflammation and septic shock formation (sepsis 3) [2]. Sepsis 3 is further complicated by persistent hypotension requiring vasopressors and is accompanied by elevated blood lactate levels (>2 mmol/L). Unless these patients are rapidly resuscitated, they can enter a pathologic state where they develop multi-organ dysfunction that can be fatal.

Endotoxin has been considered as one of the therapeutic targets for the treatment of sepsis and septic shock for a long time. The neutralization of blood endotoxin with agents such as polyclonal or monoclonal antibodies has been investigated. However, these approaches did not become clinically available. To remove endotoxin from the blood circulation with a medical device was another approach.

In 1994, we developed a selective endotoxin removal column (PMX) which contains polymyxin B covalently bound fibrous adsorbents [3]. Polymyxin B is a polycationic antibiotic which binds the lipid A portion of endotoxin and neutralizes its toxicity. Lipid A is the toxic moiety of endotoxin with a conserved structure among Gram-negative bacterial species and strains. Therefore, polymyxin B as a ligand is expected to bind many kinds of endotoxin from Gram-negative bacteria. The intravenous use of polymyxin B is contraindicated due to its nephrotoxicity and neurotoxicity [4,5]. So, polymyxin B was covalently immobilized as a ligand on the surface of a fibrous material. Extracorporeal hemoperfusion with PMX (PMX-HP) was expected to act to adsorb endotoxin from blood circulation.

PMX-HP has been clinically applied since 1994 in Japan. Currently, it is clinically available in some countries in Europe, Asia and North America. The first multicenter pilot-controlled study in six centers in Europe in 2005 confirmed the safety of PMX-HP and suggested the possibility of improving hemodynamic status and cardiac function [6]. After that, the results of three multicenter randomized controlled clinical trials (RCTs) in Italy, France and North America were published in 2009, 2015 and 2018, respectively. However, they failed to show a survival benefit in 28 days. Recently, a cohort study using a large clinical data base from the Japanese payment system DPC (diagnosis procedure combination) has suggested the improvement of mortality rate.

The endotoxin adsorption capacity in vitro and the effectiveness in animal experiments in vivo have been re-evaluated with PMX-HP. The immunostimulatory effects and the cellular elements inducing immunomodulatory effects and anti-apoptotic effects with PMX-HP have been demonstrated. However, the mechanism of action of PMX-HP is not fully understood.

In this article, we review the findings about PMX-HP through the last quarter century of clinical applications for sepsis and septic shock patients. We also discuss the history of important discoveries in endotoxin research (Table 1). We will review the evidence and describe the consequences and future directions for PMX-HP.

## 2. Historical Overview of the Anti-Endotoxin Strategy for the Treatment of Septic Shock 

The identification of the structure and function of bacterial endotoxin was one of the crowning accomplishments of the age of discovery in the late 19th century. The advances in the discipline of microbiology were central to the widespread acceptance of the “germ theory” of disease. Disease causation by microscopic organisms was largely unknown and unaccepted at the beginning of the 19th century but was axiomatic in science as a major cause of illness and death by the end of the century. Vague concepts and terms such as “miasma” or “contagion” gradually gave way to specific, careful, testable, scientific methodologies with adequate controls, sterile technique, and attention to the details to generate reproducible results. This led to generations of microbiologists from around the globe working, more or less, together in describing microorganisms in the laboratory. This requirement for careful, reproducible, results set the standard for scientific progress still used up to the present day.

The cumulative work of such scientists as Koch, Pasteur, Panum, and Klebs made tremendous contributions to microbiology. They demanded that the method devised by Koch in the laboratory be followed to confirm disease causation. This is now named Koch’s postulates. There are four elements: (1) the pathogenic organism is present when the disease is present; (2) the disease is absent when the organism is no longer present (3); the disease can be reproduced in experimental animal models; and (4) the causative pathogen can be re-isolated from and grown again from the animal model. Koch’s postulates do not always work [7], but it is still the gold standard.

One of the most important discoveries was made in 1892, when one of Koch’s favorite students, Richard Pfeiffer (1858–1945) [8], is credited for first describing the bacterial endotoxin. Exotoxins had been recognized and described earlier, but the endotoxin was different than the tetanus toxin or diphtheria toxin. These exotoxins are proteinaceous, excreted into the extracellular space and they were heat-labile. Endotoxins are highly heat stable.

When endotoxin is removed and isolated from other cell wall materials, it accounts for about 70% of the cell wall. Another major discovery in diagnostic microbiology occurred in 1884 when a Danish physician named Hans Christian-Gram (1853–1939) described a colorimetric method to distinguish enteric bacteria with endotoxin in their cell wall now named the Gram stain. Bacteria with endotoxin in their cell walls stain pink when fixed on a slide and decolorized with alcohol. These are known as Gram-negative bacteria (for example, *Pseudomonas aeruginosa*). In contrast many other bacterial species such as *Staphylococcus aureus* or *Streptococcus pyogenes* hold onto the dye and stain dark blue.

This easy method of distinguishing bacteria essentially conveniently separates the pathogenic bacterial species into two forms named either Gram-negative or Gram-positive bacteria. It is still used today as a rapid method to distinguish bacterial pathogens.

## 3. Could Bacterial Endotoxin Be a Therapeutic Target for Treating Gram-Negative Sepsis with Antibodies?

It is possible to raise antibodies—either polyclonal or monoclonal antibodies—as a potential treatment for Gram-negative sepsis or septic shock. Both strategies have been tried in large multicenter clinical trials. The core glycolipid nature of bacterial endotoxin is immunogenic and polyclonal antibodies can be raised from banked plasma from blood donors. The inner core of endotoxin is a fairly uniform structure, with Lipid A and a number of highly conserved, immunogenic epitopes.

This strategy was put to a test in the 1980s, when a number of large clinical trials were conducted to determine if it would be possible to block the pathogen invasion by giving high titer, polyclonal antiserum. These studies generated a mixture of results, but no convincing evidence of clear benefit [9,10,11]. A number of studies with monoclonal antibodies to the core structure of Lipid A and monoclonal agents to the inner core of bacterial endotoxin also failed to significantly provide benefit. This approach has been largely abandoned until better detection methods or improved and genetically engineered antibodies become available [12].

## 4. Designing of Polymyxin B Immobilized Fiber Column (PMX)

**Figure 1:**
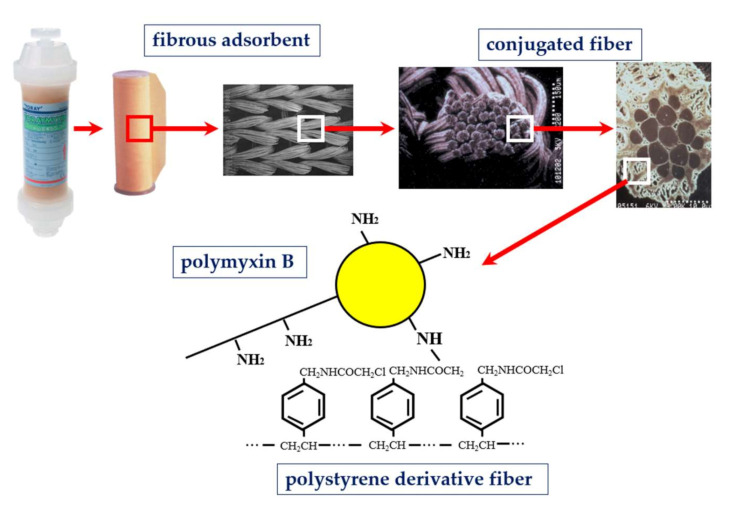
Structure of polymyxin B immobilized fiber column.

**Figure 2:**
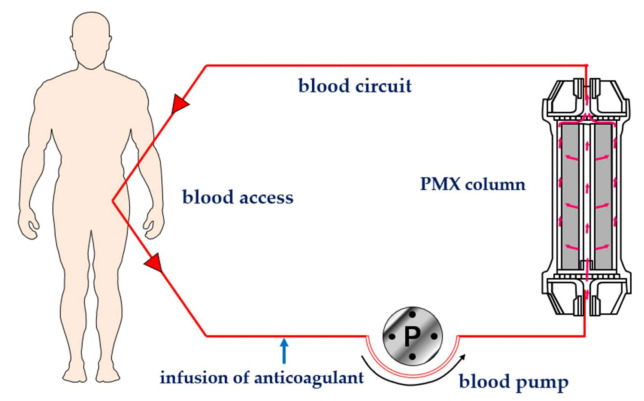
Schematic diagram of hemoperfusion with PMX (PMX-HP). PMX: polymyxin B immobilized fiber column.

Polymyxin B was covalently bonded on the surface of polystyrene-derivative fibers using primary amino group of diaminobutyric acid residue [3] (Figure 1). Immobilized polymyxin B molecules were expected to bind the lipid A portion of the endotoxin via ionic and hydrophobic interactions. Covalently fixed polymyxin B does not leak out into the blood stream. Thus, it enabled one to allow the clinical application without the known toxic effects of polymyxin B. The PMX-HP procedure is practiced through a whole blood circulation at a blood flow rate of 80 to 120 mL/min (Figure 2). As an anti-coagulant, unfractionated heparin is available. In Japan, the protease inhibitor Nafamostat mesilate is commonly used for its short half-life.

## 5. Revisiting the Endotoxin Adsorption Capacity In Vitro and In Vivo Settings

### 5.1. In Vitro Endotoxin Removal Experiments with PMX

We evaluated the endotoxin adsorption capacity for PMX (type: PMX-20R) in vitro settings [13]. LPS (*Escherichia coli* 0111: B4)-spiked bovine serum (1.5 l) was perfused in PMX for 4 h at 100 mL/min. The initial 10 ng/mL LPS concentration routinely decreased from 10 to 2–3 ng/mL after column perfusion. Therefore, the total amount of endotoxin removal was about 12 μg. In our previous test, when LPS-spiked bovine serum was perfused through PMX, the concentration reached to the adsorption equilibrium in 2 to 3 h. This latest result was well reproduced our previous study. PMX column was also perfused with 0.5 L of pooled EDTA anti-coagulated whole blood spiked with 100 μg of FITC-labeled *E. coli* O111:B4 LPS (200 ng/mL) for 2 h at a flow rate of 100 mL/min. After perfusion, bound LPS was eluted. Based on the fluorescence measurements, PMX tested bound a mean of 20 μg LPS. The endotoxin adsorption in PMX was confirmed in the whole blood hemoperfusion experiment.

Yamashita et al. sought how long the adsorption capacity of PMX could be maintained before getting to the saturated adsorption [14]. LPS was continuously infused into the reservoir with bovine serum so that the LPS concentration could be increased with time. A perfusion test with PMX (type: PMX-01R) or with a blood tubing alone as a sham-control was practiced. The changes of LPS concentration in the reservoir was always lower in the PMX test than in a control through 24 h. This result indicated that the adsorption capacity of PMX does not reach the saturation point even after a 3 h perfusion. If the adsorption equilibrium does not occur, a longer duration use of PMX more than 2 to 3 h could be suggested.

### 5.2. Animal Experiments

Iba et al. examined the effectiveness of PMX using a non-hypotensive rat sepsis model by intravenous live *E. coli* injection [15]. Wistar rats were assigned to two groups of PMX-HP and a dummy column hemoperfusion group (*n* = 7 in each group). Hemoperfusion was continued for 3 h. Organ damage markers such as ALT, LDH and BUN were lower in PMX-HP group. The levels of proinflammatory cytokines such as IL-6, TNF-α and IL-1β were significantly lower in the PMX-HP group than in the control group. Microscopic examination of mesenteric microcirculation was better maintained in PMX-HP group. The survival rate was better in PMX-HP group (93%) than in the control group (57%, *p* = 0.03).

Yeh et al. investigated the effectiveness of PMX-HP on microcirculation using a septic pig model of fecal peritonitis [16]. PMX-HP was continued for 2 h. The perfused small vessel density and tissue oxygen saturation of the ileal mucosa at 6 h were higher in the PMX-HP group than those in the sepsis group. The histologic score for the ileal mucosa was lower in the PMX-HP group than that in the control.

These two studies demonstrated that PMX-HP could attenuate microcirculatory dysfunction, tissue desaturation, and histopathologic alterations in the ileal mucosa in this septic animal model with suspected endotoxemia by removing blood endotoxin. These animal experiments could support to indicate PMX-HP for sepsis and septic shock patients with endotoxemia.

## 6. Clinical Outcome with PMX Indication

### 6.1. Multicenter Randomized Controlled Study

The EUPHAS study in Italy was the first multicenter randomized controlled study with PMX-HP [17]. The targeted population was severe sepsis and/or septic shock patients who underwent emergency surgery for intra-abdominal Gram-negative infections. PMX-HP added to conventional therapy significantly improved mean arterial pressure and vasopressor requirement and reduced 28-day mortality by 32% (11/34 patients) in the PMX-HP group and 53% (16/30 patients) in the conventional therapy group. However, the number of the enrolled patients was small, and the study was terminated early because of a statistically significant reduction of mortality in PMX-HP group. It was declared unethical to deprive a potentially beneficial therapy to a group of patients that carry high mortality rates. This invited some criticism and failed to give a definitive answer.

The ABDO-MIX study in France was a prospective, multicenter, randomized controlled trial to test whether PMX-HP reduces mortality and organ failure in peritonitis-induced septic shock from abdominal infections. The primary outcome was 28-day mortality [18]. The mortality was 27.7% (33/119) in the PMX-HP group and 19.5% (22/113) in the conventional group (*p* = 0.14). It demonstrated a non-significant increase in mortality and no improvement in organ failure with PMX-HP. The same result as EUPHAS by enrolling a similar patient population was expected, but ABDO-MIX gave a conflicting result.

Some speculations were pointed out why ABDO-MIX could not demonstrate the same result as EUPHAS. The observed 28-day mortality rate in the control group was 19.5% in ABDO-MIX and 53.3% in EUPHAS. ABDO-MIX may not have enrolled a critically sick patient for that sample size. Secondly, only 81 of the 119 treated patients (68%) completed the two scheduled sessions of PMX-HP for the column clotting and hemodynamic instability. All patients in EUPHAS had completed the two planned sessions. These two studies could not give a definitive answer to prove the effectiveness of PMX-HP for the treatment of septic shock. Further well-designed multicenter randomized controlled studies would be needed.

The EUPHRATES trial is the most recent clinical study with PMX-HP in North America [19]. The objective of this multicenter, randomized, blinded, sham-controlled trial was to test whether adding PMX-HP to conventional medical therapy improves survival compared with conventional therapy alone among patients with septic shock and high endotoxin activity value (EA value) with endotoxin activity assay (EAA).

They enrolled 450 adult critically ill patients with septic shock and EA value of 0.60 or higher. The primary outcome was mortality at 28 days among all patients randomized (all participants) and among patients randomized with a multiple organ dysfunction score (MODS) of more than 9. Among eligible 450 patients, the survival rate of the PMX treated group was 37.7% (84/223) and the control group was 34.5% (78/226). There was no significant difference in mortality at 28 days, and in the population with a MODS of more than 9, the PMX group was 44.5% (65/146) and the control group was 43.9% (65/148).

Regarding the secondary and exploratory end point analyses, the change of mean arterial pressure (MAP) in day 3 was significantly higher than the control group both in all patients’ population and in patients with MODS more than 9.0 (*p* = 0.02). Mechanical ventilation free days (VFD) to day 28 was significantly longer in PMX-HP group than the control group in patients with MODS more than 9.0 (*p* = 0.02). Investigators reported the possible reasons PMX-HP failed to improve survival. For the patients who have overwhelming blood endotoxin burden, the dose and duration of PMX-HP as applied in this trial may have been insufficient to significantly reduce the endotoxin burden.

Klein et al. did a post hoc analysis and evaluated the impact of PMX-HP in patients with septic shock and an EA value measured between 0.6–0.89 [20]. At 28 days, 26.1% patients (23/88) in the PMX-HP group died versus 36.8% (39/106) in the control group. The absolute mortality reduction was 10.7%. The 28-day survival time in the PMX-HP group was significantly longer than for the control group. PMX-HP group compared with the control group showed greater change in mean arterial pressure (MAP) and ventilator free days (VFD).

The study by Romashin et al. gave the theoretical reasons for this post hoc analysis [13]. When EA values are greater than 0.9, endotoxin mass concentrations can be much greater than 4 ng/mL. This means that in a total whole blood volume of 5 L with endotoxin equally distributed between cells and plasma, a total blood load greater than 20 μg could be achieved. If endotoxin is distributed additionally into the extracellular space (10 L), then the total extracellular endotoxin load could be significantly higher than the adsorption capacity of a single PMX-HP. In the dose–response curve of endotoxin burden (LPS in ng/mL, *y* axis) and EA value (*x* axis), it was shown that above an EA value of 0.9 (corresponding to >4 ng/mL of LPS) the curve exhibits an asymptotic behavior and thus cannot be used to quantitate LPS levels in this range. The patients with an EA value more than 0.9 may have high burden of endotoxin and may not be adequate to enroll in the current EUPHRATES protocol. Post hoc analysis gave the hypothesis-generating results. The TIGRIS multicenter randomized controlled trial in the US is ongoing to prove this hypothesis.

### 6.2. Systematic Review with Meta-Analysis for PMX-HP

Several studies have been undertaken in the past 10 years to examine the efficacy of PMX-HP. Chang T et al. included a total of 17 trials [21]. The pooled risk ratio for overall mortality was 0.81 (95% (CI), 0.70–0.95), favoring the PMX-HP group (*p* = 0.007). The included studies were stratified into three groups based on the mortality rates of the conventional treatment group: low-risk group (mortality rate < 0.3), intermediate-risk group (0.3–0.6), and high-risk group (>0.6). Subgroup meta-analysis depending upon the risk stratification revealed a significant reduction of mortality in the intermediate-risk group (risk ratio, 0.84; 95% (CI), 0.77–0.92) and high-risk group (risk ratio, 0.64; 95% (CI), 0.52–0.78), but not in the low-risk group (risk ratio, 1.278; 95% (CI), 0.888–1.839). They concluded that PMX-HP may reduce mortality in patients with severe sepsis and septic shock in specific disease severity subgroups.

Li et al. included 13 studies in the meta-analysis [22]. The use of PMX-HP could reduce overall mortality (relative risk (RR) 0.68, 95% confidence interval (CI) 0.51–0.91, *p* = 0.01). Subgroup analysis also suggested the mortality rate of patients in Acute Physiology and Chronic Health Evaluation (APACHE II) scores < 25 group (RR 0.64, 95% (CI) 0.52–0.78, *p* < 0.0001) and sepsis group (RR 0.48, 95% (CI) 0.32–0.72, *p* = 0.0003) significantly decreased after PMX-HP treatment. They found a beneficial effect of PMX-HP for non-sicker patients stratified by APACHE II, as opposed to the Chang T et al. study. They insisted it might be more reasonable to stratify disease severity subgroups by APACHE II scores rather than the mortality rates of the conventional treatment group.

Terayama et al. selected seven RCTs comparing PMX-HP with conventional therapy on the outcome of mortality in patients with severe sepsis or septic shock [23]. PMX-HP therapy was associated with lower mortality (risk ratio, 0.65; 95% confidence interval (CI), 0.47–0.89; *p* = 0.007; I^2^ = 72%). Meta-regression analysis revealed a significant negative slope between effect size of PMX-DHP therapy and baseline mortality rate in individual studies (*p* = 0.003), suggesting the probability of a beneficial effect with PMX-HP increased with increasing baseline risk.

Fujii et al. included six RCTs and derived a precisely opposite conclusion [24]. The pooled risk ratio (RR) for 28-day mortality associated with PMX-HP was 1.03 (95% confidence interval (CI) 0.78–1.36; *I*^2^ = 25%; *n* = 797). They concluded that there is currently insufficient evidence to support the routine use of PMX-HP to treat patients with sepsis or septic shock. They included three same studies as Terayama et al. study and newly included three studies, in which two studies were positive and one was a negative study of the EUPHRATES trial [19]. So, it is estimated that the negative result of large scale RCTs had a much greater impact.

The results of systematic review with meta-analysis could not give a definitive answer. The conclusion derived was changed depending on the studies they could include for the analysis. Further rigorous RCTs targeting the pre-defined adequate patients who are likely to benefit from PMX-HP are warranted to define the clinical role of PMX-HP.

### 6.3. Cohort Study Using a Large Clinical Database

Iwagami et al. utilized the DPC database from 2007–2012 and assessed the survival benefit of PMX-HP in septic shock patients who received vasopressor infusion and continuous renal replacement therapy (CRRT) in the ICU [25]. Acute kidney injury (AKI) often occurrs as a complication of sepsis and is associated with high mortality in the ICU. They hypothesized that septic shock patients who required renal replacement therapy for AKI were sufficiently ill to represent the proper target population for PMX-HP. Of 3759 eligible patients, 1068 received PMX-HP. After propensity score matching, they produced a matched cohort of 978 pairs. The 28-day mortality was 40.2% (393/978) in the PMX-HP group and 46.8% (458/978) in the control group (*p* = 0.003). This large retrospective study using the DPC data base suggested that septic shock patients starting CRRT might benefit from PMX-HP.

Nakamura et al. used the dataset of the Japan Septic Disseminated Intravascular Coagulation (JSEPTIC DIC) study conducted in 40 institutions, which aimed to evaluate anti-DIC drugs in patients with severe sepsis or septic shock [26]. They investigated the potential survival benefit of PMX-HP retrospectively in this patient’s population. Of 1723 eligible patients, 522 had received PMX-HP. After propensity score matching, 262 matched pairs were obtained. The proportion of all-cause hospital mortality was significantly lower in the PMX-HP group than in the non-PMX-HP group (32.8% vs. 41.2%, *p* = 0.042).

### 6.4. Registry Study after EUPHAS Trial in Italy

The EUPHAS 2 study is a multicenter registry study for PMX-HP, and the aim was to verify the application of PMX-HP in the daily clinical practice (https://www.euphas2.eu accessed on 18 February 2021. Phase 1 in EUPHAS 2 involved 57 centers between January 2010 and December 2014, collecting retrospective data of 357 patients (297 in Europe and 60 in Asia) suffering from severe sepsis and septic shock caused by proved or suspected Gram-negative infection [27]. Septic shock was diagnosed in 305 (85.4%) patients and severe sepsis in 52 (14.6%). The most common source of infection was abdominal (44.0%) followed by pulmonary (17.6%). Gram-negative bacteria represented 60.6% of the pathogens responsible for infection. The survival rate of 28-days was 54.5% (60.4% in abdominal and 47.5% in pulmonary infection). Patients with abdominal infection treated with PMX-HP within 24 h from the diagnosis of septic shock had a 28-day survival rate of 64.5%. This number was comparable with 68% observed in the EUPHAS study [17]. There were no life-threatening adverse events related to PMX-HP and the feasibility of PMX-HP application was confirmed. The blood endotoxin (EA value) was measured in 132 out of 357 patients (37.0%). The measurement was possible in 18 out of 24 centers. A median EA value at the time 0 was 0.77 (0.69–0.90). The EA value of ≥0.6 was in 120 patients and less than 0.6 in 12 patients. This means that 90% (120/132) of the patients whose EA value was measured had a high value. Endotoxemia may be accompanied frequently in this patient’s population. Phase 2 in the EUPHAS 2 registry has been ongoing since 2015.

## 7. Host Response to PMX-HP Application

### 7.1. The Changes of Blood Endotoxin Level

The blood endotoxin concentration of the radial artery and the outlet of the PMX column was measured with endotoxin specific limulus amebocyte lysate assay (LAL) for 19 patients after a 24-h duration of PMX-HP to evaluate the endotoxin removal [28]. In 11 patients, the endotoxin level at the outlet of PMX was lower than that in the radial artery, demonstrating the removing capability at 24 h. In the other eight patients, the endotoxin level at the radial artery was already lowered to the normal limits (less than 1.1 pg/mL). In 13 of 19 (68.4%) patients whose endotoxin level decreased after PMX-HP, 6 (46%) patients died in 28 days. The APACHE II score of these six patients was extremely high, from 29 to 40. PMX-HP might have been started too late to rescue these patients from multiple organ failure. In six patients, the endotoxin level did not decrease, but stayed high at 24 h. The median radial arterial plasma endotoxin concentration for all 19 patients was 16.48 pg/mL at the initiation timing of PMX-HP. After 24 h of PMX-HP, the endotoxin level decreased to 1.857 pg/mL. The median removal rate was 74.4%.

Novelli et al. investigated the usefulness of EAA for patients after surgery to find high-risk patients and to determine the need of multiple applications of PMX-HP [29]. Thirty-eight post-surgical patients were enrolled. Seventeen patients with a high level of EA value (≥0.6) received standard therapy plus PMX-HP every 24 h to lower the EA value to less than 0.4. Seven patients required two sessions of PMX-HP, eight required three sessions, and two required four sessions. The EA value steadily decreased following PMX-HP application. As a result, all 17 patients survived for 28 days. These studies demonstrated that the blood endotoxin level could be reduced with PMX-HP. In the case that the level does not decrease by one session, the repeated application might be effective depending on the endotoxin burden of the patient.

### 7.2. Immunostimulatory Effects

The host response to infection is characterized by both proinflammatory and anti-inflammatory responses. Immune cells such as monocyte, macrophage and neutrophil can cause either acute proinflammatory signals or anti-inflammatory signals and play a central role in the host defense. Drewry et al. studied monocyte HLA-DR expression as an outcome predictor in severe sepsis and concluded monocyte HLA-DR expression might be a more accurate predictor of mortality and the acquisition of secondary infections than LPS-stimulated TNF-α production in adult medical and surgical critically ill patients. [30]. Ono et al. investigated the changes of the expression of the HLA-DR antigen on monocytes and CD16 on granulocytes with PMX-HP [31]. Thirty-four patients after emergency surgery due to intra-abdominal infection were enrolled. HLA-DR antigen expression and the intensity of CD16 antigens were markedly lower in patients with septic shock than that in sepsis patients and healthy volunteers. Significant negative correlations were observed between the severity score (APACHE II) and HLA-DR expression (%) or the intensity of the CD16 antigen. Ten septic shock patients were treated with PMX-HP. The expression of the HLA-DR antigen on monocytes and the CD16 antigen on neutrophils significantly increased after PMX-HP. HLA-DR is involved in antigen presentation to T cells, and CD16 is involved in phagocytosis and cytotoxicity by neutrophils. The blood endotoxin level was not measured, but endotoxemia was highly suspected from the background of the patients and the detection of Gram-negative bacteria.

Srisawat et al. conducted a randomized controlled trial in patients with severe sepsis or septic shock with documented elevated blood levels of endotoxin (EA value ≥0.6) [32]. They sought to evaluate the immune response with PMX-HP. Twenty-nine (29) patients received PMX-HP plus standard treatment for 2 consecutive days. Thirty patients (30) in the control group received only standard treatment. At baseline, monocyte HLA-DR expression was comparable between two groups. The median change in monocyte HLA-DR expression between day 3 and baseline was higher in the PMX-HP group than in the control group (*p* = 0.027). CD11b, which represents neutrophil activation, was not increased in the PMX-HP group, but significantly increased in the control group. The stabilization of neutrophil activation was suggested in PMX-HP. The immunomodulatory effect with PMX-HP was confirmed in the patients with documented elevated levels of endotoxin.

PMX-HP treatment could improve the immunosuppression state of the septic patients, which might lead to the improvement of outcomes. The mechanism(s) of action, whether it is derived from the result of endotoxin removal or any other action of PMX-HP, should be studied in greater detail.

### 7.3. Cellular Elements Alteration with PMX-HP

#### 7.3.1. Neutrophil

Neutrophils from septic shock patients (*n* = 18) showed a higher level of CD11b/CD64 expression and a lower level of chemokine receptor CXCR1/CXCR2 expression than those from healthy controls [33]. The mean percentage of neutrophils expressing CXCR1/CXCR2 was significantly increased after PMX-HP, whereas CD11b/CD64 expression was significantly decreased. The elevated plasma levels of cytokines (IL-6, IL-8, IL-10, and HMGB-1) were not altered with PMX-HP. The mechanism without cytokine involvement was suggested. In ex vivo experiments with heparinized sepsis and septic shock patients’ blood perfused through the PMX column, a significant reduction of neutrophil and monocyte counts was observed. Flow cytometric analysis indicated that activated neutrophils with a high expression of CD11b/CD64 and a low expression CXCR1/CXCR2 were selectively adsorbed on the PMX column compared to a sham column. The selective removal of activated neutrophils was suggested for PMX-HP to improve the abnormalities of immune function of sepsis and septic shock patients. Leukocyte adhesion on the adsorbent of PMX was reported by other studies [34,35]. However, the mechanism of a selective adhesion of neutrophil is left to be studied.

#### 7.3.2. T-LYMPHOCYTE

CD4^+^CD25^+^Foxp3+ regulatory T cell (Treg) produces large quantities of the anti-inflammatory cytokines IL-10 and transforming growth factor (TGF-β) and suppress interferon-γ (IFN-γ) production. Treg plays a major role in the immunosuppressive stage of late sepsis. Ono et al. investigated the role of Treg in septic shock patients, and the effect of PMX-HP on reducing Treg [36]. The percentage of Tregs in the CD4+ T-cell population and the serum IL-6 and IL-10 levels were significantly higher among patients with septic shock compared with those without septic shock. PMX-HP significantly decreased the number of Tregs, as well as the serum cytokine levels. However, the reason why PMX-HP decreases the number of Tregs with PMX-HP is not yet explained.

#### 7.3.3. Apoptotic Cell

Apoptosis (programmed cell death) and necrosis are the way of cell death. Enhanced apoptosis of immunocompetent cells such as B cells and CD4+T cells has been implicated in sepsis-associated immunosuppression. It has been reported that apoptosis was induced not only in immunocompetent cells, but also in parenchymal tissues as seen in the cases of sepsis. Cantaluppi et al. studied the apoptosis of renal tubular and glomerular cells involved in the development of acute kidney injury (AKI) associated with sepsis [37]. The patients’ plasma collected before PMX-HP and 72 h later was cocultured with human renal tubular cells. Fas (CD95) ligand was highly expressed on the renal tubular cell surface and decreased after 72 h in the PMX-HP group. In the control group treated with the conventional therapy, Fas (CD95) expression was not reduced even in 72 h. Organ injury scores of sepsis related organ failure accessment (SOFA) and risk, injury, failure loss end- stage kidney disease (RIFLE) were all significantly reduced after PMX-HP. PMX-HP reduced the proapoptotic activity of the plasma of septic patients on cultured renal cells. As the plasma levels of endotoxin were significantly reduced, it was suggested that there was a strong correlation between the reduced levels of endotoxin and the plasma-induced tubular apoptosis. A putative protective effect of PMX-HP was indicated for the early prevention of kidney injury.

Ito et al. studied the pathological assessment of isolated lungs, livers, and kidneys with a rat CLP (cecal ligation and puncture) model [38]. As a control, they practiced a hemoperfusion with a sham column. The number of apoptotic cells in kidney tubular cells was significantly lower in the PMX-HP group compared with the sham perfusion group. In other organs, the reduction of apoptotic cell counts was not detected. It was estimated that endotoxin was highly involved to induce apoptosis in this bacterial infection model, but the exact reason was not clear. Mitaka et al. also reported that PMX-HP might protect against acute kidney injury not only by inhibiting the NF-kB signaling pathway but also by preventing renal tubular cell apoptosis in a rat model [39].

### 7.4. Future Direction of PMX-HP, Endotoxin Removal with Cellular Alteration of Immune Cells

The directions of a precision medicine, a new indication and the mechanism of action for PMX-HP should be sought out. The diagnosis of endotoxemia is an important issue in selecting a patient who is likely to benefit from PMX-HP. Endotoxin-specific LAL assay is available in Japan [40]. EAA demonstrated a significant correlation with the severity of sepsis patients [41,42]. However, the diagnosis of endotoxemia is still controversial. It is required to establish the diagnostic method for endotoxemia which is relevant to the clinical condition.

PMX-HP has been applied for the treatment of the acute exacerbation of idiopathic pulmonary fibrosis (AE-IPF) since 2006 [43,44]. Japanese guidelines for IPF published in 2017 mentioned that the evidence was scant and suggested that patients with AE-IPF should not be treated with PMX-HP, but that this therapy might be a reasonable option in a minority of patients [45]. A RCT for AE-IPF with PMX-HP has not yet been carried out. Many studies have demonstrated the improvement of oxygenation and mortality. The elimination of activated neutrophil with PMX-HP has been suggested as the effective mechanisms for AE-IPF [35]. Acute exacerbation is most likely triggered by an acute event. Despite the large number of studies to date, definitive conclusions about the role of infection in IPF have not yet been made. Endotoxemia should be evaluated.

During the 2009 H1N1 pandemic, PMX-HP demonstrated the improvement of oxygenation index (PaO_2_/FiO_2_) for the patients with the severe respiratory failure [46,47]. PMX-HP application for the critically ill COVID-19 patients has been reported [48,49,50,51]. Recently, it was reported that EA value with EAA was high in COVID-19 patients [52]. Critical illness due to viral infection might be a reasonable therapeutic target for PMX-HP. Further study is required to indicate PMX-HP for a new indication.

A mechanism of action of PMX-HP to improve the organ dysfunction is not well understood. The results of endotoxin removal and/or the cellular depletion accompanied with cellular alteration of immune cells should be studied to clarify the mechanisms.

## 8. Conclusions

PMX-HP has been safely used for the treatment of septic shock since 1994. The survival benefit of this therapy is still suggestive by the cohort study using a large clinical database. A currently ongoing TIGRIS multicenter randomized controlled trial is expected to prove the evidence for the treatment of septic shock patients with endotoxemia who are likely to benefit from PMX-HP. PMX-HP is not only endotoxin removal, but also an immunomodulatory device. Further study is required to clarify the mechanism of action of PMX-HP and to apply for a good patient population.

## Figures and Tables

**Table 1:** Historical Milestones.

Event	Year(s)	Major Findings
Discovery of microorganisms	1676	Robert Hooke and Antoni van Leeuwenhoek independently discover living microorganisms by careful microscopy using lenses to identify microbes.
Proving the germ theory of disease	1860s	Louis Pasteur (1822–1864) and Robert Koch (1843–1910) demonstrate that microorganisms in infected tissues directly cause tissue injury. Organisms can be transmitted between animals and humans.
Ignas Semmelweis	1850s	Semmelweis (1818–1865) proves in 1847 that microbial pathogens can be transmitted by the hands of doctors and cause potentially lethal puerperal fever; hand hygiene prevents this from happening.
Discovery of Endotoxin	1892	Robert Koch and his colleague Richard Pfeiffer first prove that about 70% of the cell wall of Gram-negative bacteria is protease resistant and lipid sensitive material. They demonstrate that purified endotoxin injected intravenously is lethal to laboratory animals, forming Koch’s Postulates.
Gram’s stain aids to detect and define bacteria	1884	Hans Christian Gram (1853–1938) first develops a method to rapidly classify and identify bacteria as either Gram-positive or Gram-negative by differential staining and microscopy.
Polymyxin B bound hemofilters remove endotoxin	1994	Tohru Tani and Hisataka Shoji, et al. develop the cationic filters which will bind to circulating endotoxin and remove endotoxin from the circulation and can rescue patients from endotoxemia.
The 3-dimensional structure of endotoxin is solved	2000s	Beutler and colleagues define the structure of TLR 4 as the molecular receptor for endotoxin and how Lipid A, core glyco-lipid and MD2 interact to signal the presence of endotoxin.
Clinical trials of endotoxin filters, mono-clonal antibodies	2013–2020	Clinical trials are underway to determine if these strategies can improve outcome.
